# Factors associated with SARS-CoV-2 and community-onset invasive *Staphylococcus aureus* coinfection, 2020

**DOI:** 10.1017/ash.2023.342

**Published:** 2023-09-29

**Authors:** Kelly Jackson, Sydney Resler, Joelle Nadle, Susan Petit, Susan Ray, Lee Harrison, Ruth Lynfield, Kathryn Como-Sabetti, Carmen Bernu, Ghinwa Dumyati, Marissa Tracy, William Schaffner, Holly Biggs, Isaac See

## Abstract

**Background:** Previous analyses describing the relationship between SARS-CoV-2 infection and *Staphylococcus aureus* have focused on hospital-onset *S. aureus* infections occurring during COVID-19 hospitalizations. Because most invasive *S. aureus* (iSA) infections are community-onset (CO), we characterized CO iSA cases with a recent positive SARS-CoV-2 test (coinfection). **Methods:** We analyzed CDC Emerging Infections Program active, population- and laboratory-based iSA surveillance data among adults during March 1–December 31, 2020, from 11 counties in 7 states. The iSA cases (*S. aureus* isolation from a normally sterile site in a surveillance area resident) were considered CO if culture was obtained <3 days after hospital admission. Coinfection was defined as first positive SARS-CoV-2 test ≤14 days before the initial iSA culture.  We explored factors independently associated with SARS-CoV-2 coinfection versus no prior positive SARS-CoV-2 test among CO iSA cases through a multivariable logistic regression model (using demographic, healthcare exposure, and underlying condition variables with P<0.25 in univariate analysis) and examined differences in outcomes through descriptive analysis. **Results:** Overall, 3,908 CO iSA cases were reported, including 138 SARS-CoV-2 coinfections (3.5%); 58.0% of coinfections had iSA culture and the first positive SARS-CoV-2 test on the same day (Fig. 1). In univariate analysis, neither methicillin resistance (44.2% with coinfection vs 36.5% without; *P* = .06) nor race and ethnicity differed significantly between iSA cases with and without SARS-CoV-2 coinfection (*P* = .93 for any association between race and ethnicity and coinfection), although iSA cases with coinfection were older (median age, 72 vs 60 years , P<0.01) and more often female (46.7% vs 36.3%, P=0.01).  In multivariable analysis, significant associations with SARS-CoV-2 coinfection included older age, female sex, previous location in a long-term care facility (LTCF) or hospital, presence of a central venous catheter (CVC), and diabetes (Figure 2).  Two-thirds of co-infection cases had ≥1 of the following characteristics: age > 73 years, LTCF residence 3 days before iSA culture, and/or CVC present any time during the 2 days before iSA culture. More often, iSA cases with SARS-CoV-2 coinfection were admitted to the intensive care unit ≤2 days after iSA culture (37.7% vs 23.3%, P<0.01) and died (33.3% vs 11.3%, P<0.01). **Conclusions:** CO iSA patients with SARS-CoV-2 coinfection represent a small proportion of CO iSA cases and mostly involve a limited number of factors related to likelihood of acquiring SARS-CoV-2 and iSA. Although CO iSA patients with SARS-CoV-2 coinfection had more severe outcomes, additional research is needed to understand how much of this difference is related to differences in patient characteristics.

**Figure:**
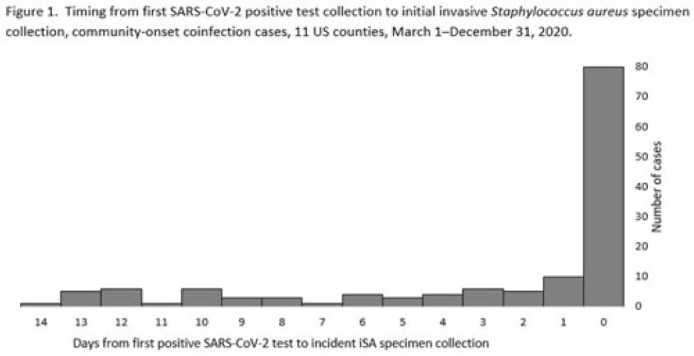

**Figure:**
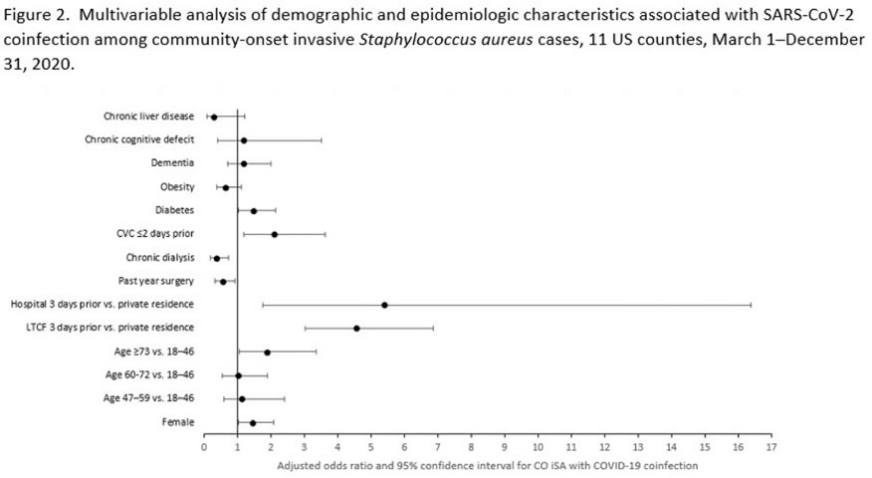

**Disclosures:** None

